# Disproportionality Analysis of Breast Cancer-Related Reports Across Vitamin K Homologs in the FDA Adverse Event Reporting System

**DOI:** 10.3390/ph19081181

**Published:** 2026-07-28

**Authors:** Shinsuke Miyazawa, Yoshihiro Uesawa

**Affiliations:** 1 Department of Community Health Care and Sciences, Meiji Pharmaceutical University, 2-522-1 Noshio, Kiyose-shi 204-8588, Tokyo, Japan; 2 Department of Medical Molecular Informatics, Meiji Pharmaceutical University, 2-522-1 Noshio, Kiyose-shi 204-8588, Tokyo, Japan; uesawa@my-pharm.ac.jp

**Keywords:** breast cancer, vitamin K, quinone, FAERS, reporting disproportionality

## Abstract

**Background/Objective:** Vitamin K (VK) comprises a family of quinone compounds with potential involvement in cell death-related pathways through their redox properties. However, consistent findings have not been obtained regarding the clinical significance of VK in breast cancer (BC). Thus, we used the FDA Adverse Event Reporting System (FAERS) to examine the co-reporting patterns of BC-related reporting terms among VK-related reports. **Methods:** FAERS reports from 2004 Q1 to 2024 Q3 were analyzed using narrow-scope Preferred Terms from two BC-related Standardized MedDRA Queries. Reporting odds ratios (RORs), proportional reporting ratios (PRRs), Fisher’s exact tests, and expected co-report counts under independence were calculated for VK overall and individual homologs, followed by exploratory comparisons with other quinone-containing compounds. An INDI-based sensitivity analysis excluded reports with BC-related indication terms. **Results:** Among 32,156 VK-related reports, 136 were BC-related. VK overall showed significantly lower reporting disproportionality (ROR, 0.486; 95% confidence interval [CI], 0.411–0.575). The predefined criteria for lower reporting disproportionality were also met for phytomenadione (VK1), menatetrenone (MK-4), the integrated VK2 category, and menadione (VK3). For VK3, 2 co-reports were observed compared with 13.59 expected (ROR, 0.183; 95% CI, 0.053–0.631). In the INDI-based sensitivity analysis, the lower reporting pattern remained for VK overall and VK1, was retained but borderline for the integrated VK2 category, and was not retained for MK-4 or VK3. **Conclusions:** In FAERS, BC-related terms were infrequently co-reported with VK-related reports (ROR < 1), with this pattern being partially retained in the INDI-based sensitivity analysis. This reporting pattern does not indicate reduced incidence, a protective or antitumor effect, or any causal relationship. However, it may reflect the indication structure of the comparator reporting population and other sources of reporting biases. These descriptive observations are presented solely to generate hypotheses for future mechanistic research and analytical epidemiological studies with more rigorous confounding control.

## 1. Introduction

Vitamin K (VK) comprises a family of fat-soluble compounds with a quinone structure based on a 2-methyl-1,4-naphthoquinone core. According to differences in the side chain at the 3-position, VK is classified into phytomenadione (VK1), microbially derived menaquinone (VK2), and the synthetic side chain-free form of menadione (VK3) [1,2]. Because these VK homologs share a naphthoquinone core, their ability to exhibit distinct redox behavior depending on the reduction pathway is considered an important factor influencing the diversity of their physiological and pharmacological actions.

Classically, VK functions as an essential cofactor in the gamma-carboxylation of blood coagulation factors, a component of the canonical vitamin K cycle [3]. Under physiological conditions in this pathway, VK functions as the two-electron-reduced hydroquinone form, which is not considered to involve reactive oxygen species (ROS) production. Recently, VK has been reported to function as an antioxidant that suppresses lipid peroxidation through an NAD(P)H-dependent reduction pathway mediated by ferroptosis suppressor protein 1 (FSP1) [4]. This pathway also converts VK to its hydroquinone form via two-electron reduction, and it is considered to function in a cytoprotective manner because it does not generate ROS.

Conversely, VK homologs differ in their redox behavior and ROS-generating capacity. In particular, VK3, which lacks a side chain, is readily subjected to one-electron reduction mediated by enzymes such as NADPH-cytochrome P450 reductase. The resulting semiquinone radical continuously generates ROS through redox cycling [5,6]. This ROS production can lead to strong antitumor activity through mitochondrial dysfunction and apoptosis induction. However, VK3 also exhibits nonselective cytotoxicity toward normal cells, making it unsuitable for clinical application despite its anticipated antitumor effects [5,6].

Based on these considerations, VK2 homologs, particularly menatetrenone (MK-4), have received extensive basic research attention from the perspectives of relative safety and pharmacological characteristics [5,7]. For example, in hematologic cancer models using HL-60 cells, multiple reports indicated that MK-4 exerts antitumor effects through ROS-associated apoptosis induction [8,9]. In contrast, in models using the triple-negative breast cancer (TNBC)-derived solid tumor cell line MDA-MB-231, MK-4 induced cellular responses distinct from those observed in hematologic cancers and nonapoptotic cell death accompanied by autophagy induction has been observed [10].

ROS are major upstream factors that induce apoptosis through mitochondrial dysfunction and caspase activation. However, ROS may also contribute to autophagic responses to oxidative stress, and the balance between cell survival and cell death may depend on the amount and duration of ROS production and the cellular background [5,6,10]. These findings suggest that the antitumor effects of VK2 could diverge into different cell death modalities depending on the cancer type and cellular context. Nevertheless, epidemiological studies assessing dietary VK intake have not yielded consistent findings for breast cancer, including differences among homologs. Prospective studies have identified associations between VK2 intake and breast cancer incidence or mortality, but the direction of these associations has been inconsistent across studies. In addition, case–control studies have suggested inverse associations between the index of nutritional quality for VK and breast cancer [11,12,13]. This discrepancy might involve redox properties arising from the quinone structure of VK, pharmacological differences among homologs, and differences in relevant conditions in actual clinical practice.

Analyses based on the FDA Adverse Event Reporting System (FAERS) database, in addition to detecting adverse-event signals, have identified specific drugs or compounds with relatively low reporting distributions for specific disease-related adverse-event terms (reporting odds ratio [ROR] < 1). These distributions have been used as a basis for the multifaceted evaluation of drug–disease relationships by comparing them with mechanistic findings suggested by existing basic research [14,15]. In oncology, report distribution analyses based on FAERS and similar databases have been conducted for sodium channel-blocking antiepileptic drugs and multiple cancer types [16], as well as digoxin in gastrointestinal cancers and hematologic malignancies [17]. Similar investigations have also been performed in multiple sclerosis for antidiabetic drugs [18] and bile acids [19]. A common feature of these reports is that the reporting distribution characteristic of ROR < 1 in FAERS is positioned as a hypothesis-generating observation regarding the drug–disease relationship by comparison with mechanistic findings established in basic research.

Concerning VK homologs, MK-4 has already been reported to induce nonapoptotic cell death accompanied by autophagy induction in MDA-MB-231 TNBC cells [10], and VK3 has been found to induce ROS-dependent cell death in breast cancer cell lines (MCF-7 and MDA-MB-231) [20,21]. Given these basic research findings, examining the reporting distribution of VK-related and breast cancer-related reports in FAERS by homolog and structure may provide descriptive information for hypothesis generation regarding previously reported differences in quinone structure, redox properties, and cellular responses.

The relationship between homolog-specific differences and clinical reporting patterns should be further explored to clarify the conflicting basic research results among VK homologs and inconsistent epidemiological findings on intake and incidence or mortality in breast cancer. Therefore, this study conducted a hypothesis-generating, descriptive analysis of the reporting disproportionality of breast cancer-related reports in FAERS, specifically focusing on VK-related reports. We also conducted homolog-specific analyses and exploratory comparisons with other quinone-containing compounds. The aforementioned mechanistic and basic research findings are provided solely as background context for hypothesis generation and are not intended to explain the reporting patterns observed in FAERS.

## 2. Results

### 2.1. Relationship Between VK-Related Reports and Breast Cancer-Related Reports

**Figure 1:**
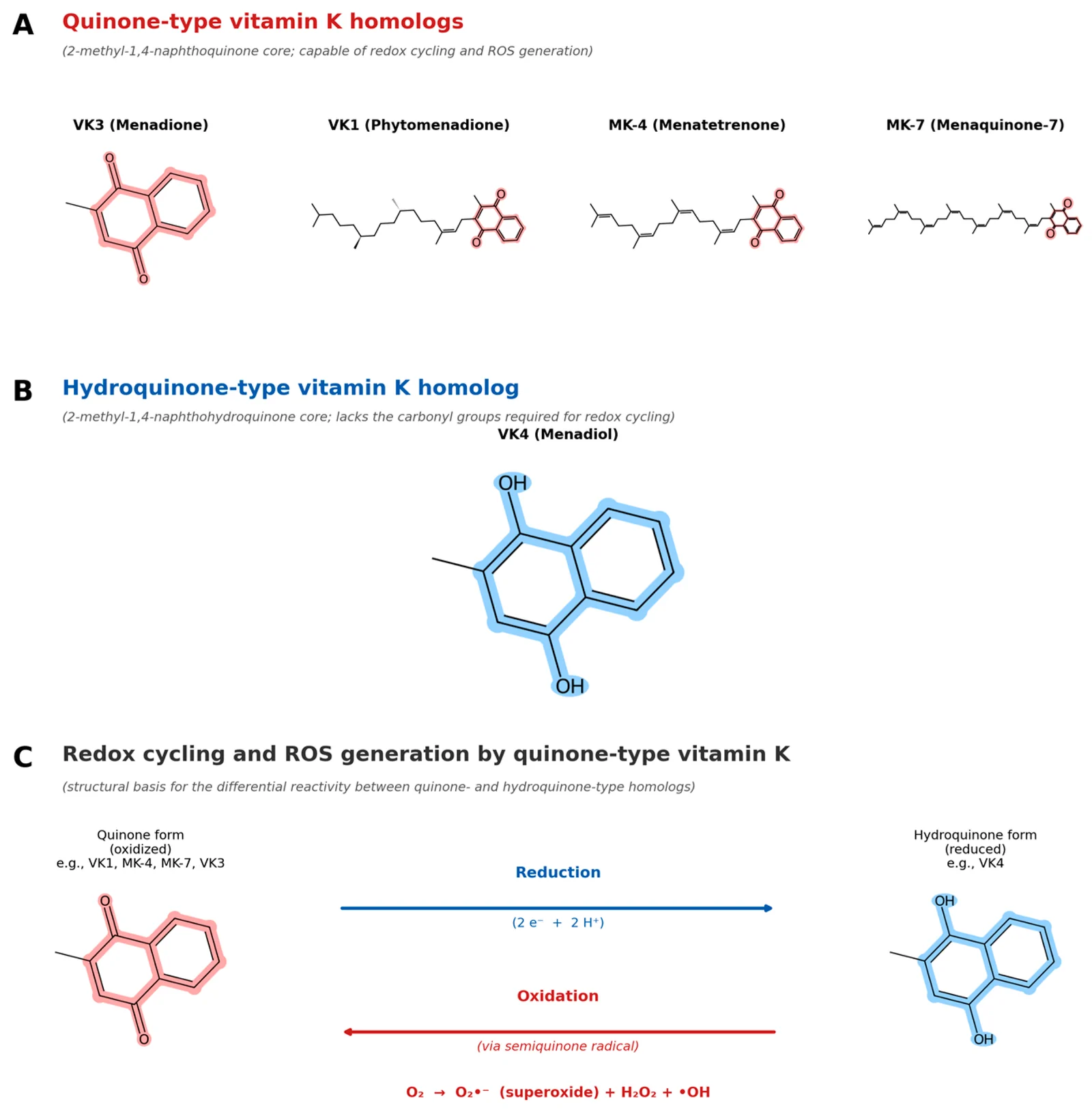
Chemical structures of VK homologs. (**A**) Quinone-type VK homologs share a common 2-methyl-1,4-naphthoquinone core (highlighted in red), with structural variation limited to the side chain at the 3-position: VK3 lacks a side chain, VK1 carries a phytyl side chain, MK-4 features a tetraprenyl side chain, and MK-7 possesses a heptaprenyl side chain. (**B**) The hydroquinone-type homolog VK4 shares the methylated naphthalene scaffold but carries hydroxyl groups instead of carbonyl groups at the 1- and 4-positions (highlighted in blue), therefore lacking the carbonyl moieties required for redox cycling. (**C**) Schematic of the redox cycling between quinone (oxidized) and hydroquinone (reduced) forms. Reduction proceeds via two-electron transfer with two protons: reverse oxidation generates a semiquinone radical intermediate that can transfer an electron to molecular oxygen to produce superoxide (O_2_^−^) and downstream reactive oxygen species (H_2_O_2_, OH). Structures were drawn using RDKit (version 2025.03.4, https://www.rdkit.org, accessed on 6 August 2025).

**Figure 2:**
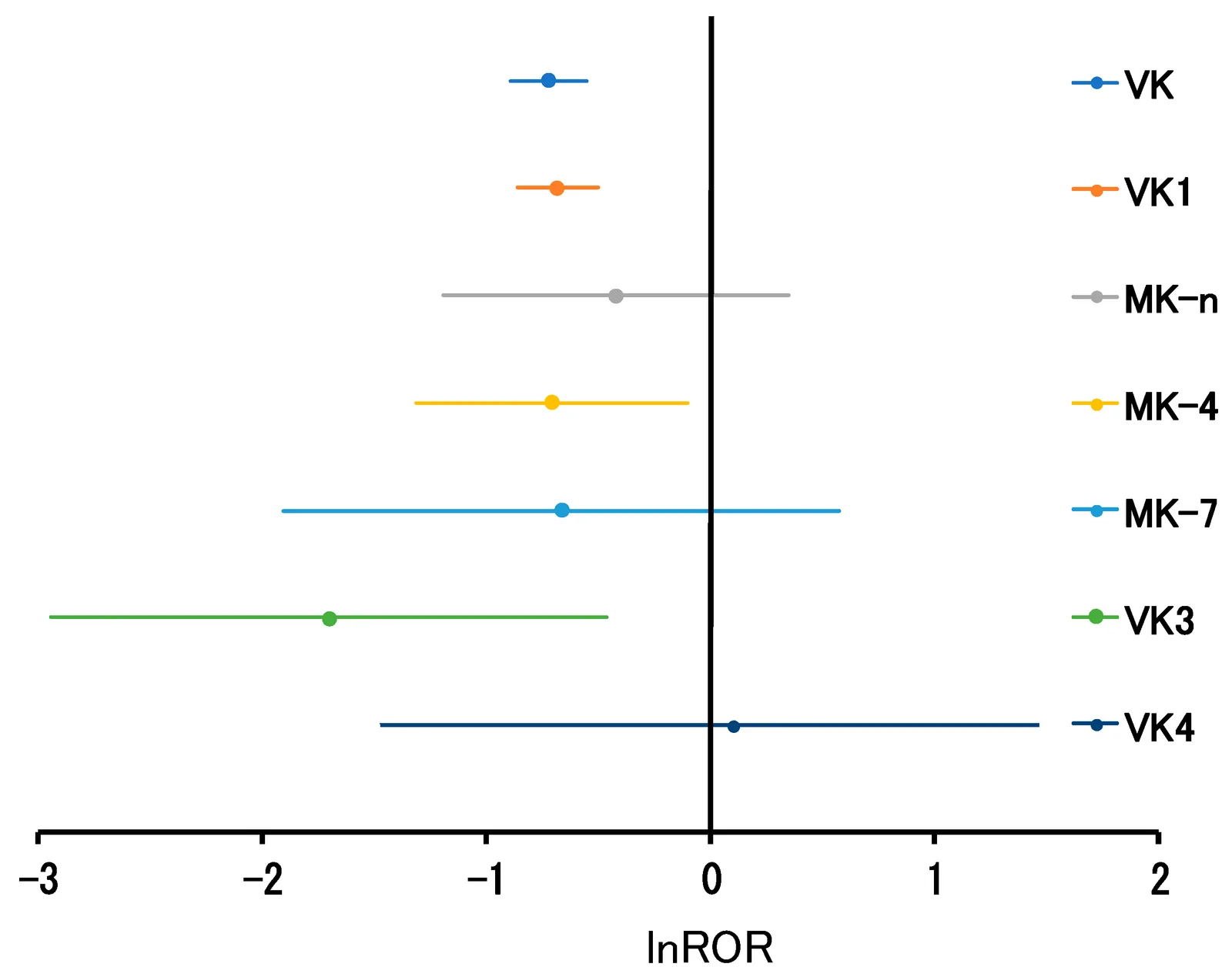
Forest plot of lnROR with 95% confidence intervals for breast cancer-related reports across VK homologs in FAERS. Horizontal bars represent 95% confidence intervals, and the vertical line at lnROR = 0 corresponds to ROR = 1 (no reporting disproportionality).

The chemical structures of the VK homologs analyzed in this study are presented in Figure 1. VK1, pooled menaquinones reported as “menaquinone” in FAERS (MK-n), MK-4, menaquinone-7 (MK-7), and VK3 all have quinone-type structures sharing a common 2-methyl-1,4-naphthoquinone core, differing only in the chain length and degree of unsaturation of the side chain. Conversely, menadiol (VK4) possesses a 2-methyl-1,4-naphthohydroquinone-type structure with hydroxyl groups at the 1- and 4-positions of the naphthalene ring, and it is the only homolog lacking carbonyl groups. The results of the analysis are presented in Table 1 and Figure 2.

The total number of FAERS reports (2004 Q1–2024 Q3) was 18,320,966, including 159,180 breast cancer-related reports. VK-related reports totaled 32,156, of which 136 were breast cancer-related reports.

Among VK-related reports, 31,845 included a single homolog. Conversely, 311 reports included multiple homologs (2 homologs, 302 reports; 3 homologs, 9 reports), accounting for fewer than 1% of all VK-related reports. The 2 × 2 analysis indicated that VK-related reports exhibited significantly lower reporting disproportionality for breast cancer-related reports (ROR, 0.486; 95% CI, 0.411–0.575; proportional reporting ratio [PRR], 0.488; *p* < 0.0001). The integrated VK2 category also showed significantly lower reporting disproportionality for breast cancer-related reports (ROR = 0.516; 95% confidence interval [CI], 0.327–0.815; PRR = 0.518; *p* = 0.0018). In the INDI-based sensitivity analysis, which excluded reports containing breast cancer-related indication terms described in the INDI table, the lower reporting disproportionality was statistically significant for overall VK-related reports, whereas the integrated VK2 category showed a borderline lower reporting pattern. This exclusion decreased the total number of analyzed FAERS reports from 18,320,966 to 16,055,201, breast cancer-related reports from 159,180 to 128,435, and VK-related reports from 32,156 to 15,178. Of the 136 breast cancer-related reports co-reported with VK in the primary analysis, 42 remained after the exclusion. The lower reporting disproportionality for overall VK-related reports was retained (Supplementary Table S5).

#### 2.1.1. Relationship Between VK1-Related Reports and Breast Cancer-Related Reports

In total, 26,632 reports were related to the quinone-type VK homolog VK1, including 117 breast cancer-related reports. Significantly lower reporting disproportionality was observed for breast cancer-related reports among VK1-related reports (ROR, 0.505; 95% CI, 0.421–0.606; PRR, 0.507; *p* < 0.0001). In the INDI-based sensitivity analysis, the lower reporting disproportionality for VK1-related reports remained statistically significant (ROR, 0.336; 95% CI, 0.239–0.472; PRR, 0.338; *p* < 0.0001) (Supplementary Table S5).

#### 2.1.2. Relationship Between VK2-Related and Breast Cancer-Related Reports

The quinone-type VK2 homologs identified in FAERS included MK-n, MK-4, and MK-7. In the integrated analysis of these VK2 homologs, 4106 VK2-related reports, including 18 breast cancer-related reports, were identified with a significantly lower reporting disproportionality (ROR, 0.516; 95% CI, 0.327–0.815; PRR, 0.518; *p* = 0.0018). In the analyses by VK2 homolog, MK-n-related reports totaled 1133, including 6 breast cancer-related reports; MK-4-related reports totaled 2436, including 10 breast cancer-related reports; and MK-7-related reports totaled 556, including 2 breast cancer-related reports. Because some reports included multiple VK2 homologs, the homolog-specific counts were not mutually exclusive. Among the homolog-specific analyses, significantly lower reporting disproportionality was observed only for MK-4-related reports (ROR, 0.494; 95% CI, 0.269–0.905; PRR, 0.496; *p* = 0.0115). MK-n had 6 observed versus 9.84 expected co-reports (ROR, 0.658; 95% CI, 0.304–1.422), whereas MK-7 had 2 observed versus 4.83 expected co-reports (ROR, 0.514; 95% CI, 0.148–1.782). Neither estimate met the predefined criteria for statistically significant reporting disproportionality because the corresponding 95% CIs included 1. In the INDI-based sensitivity analysis, the integrated VK2 category retained a borderline lower reporting disproportionality (ROR, 0.502; 95% CI, 0.256–0.984), whereas the estimates for MK-n, MK-4, and MK-7 did not meet the predefined criteria (Supplementary Table S5).

#### 2.1.3. Relationship Between VK3-Related Reports and Breast Cancer-Related Reports

The number of reports related to VK3 was 1564, of which 2 were breast cancer-related reports. Significantly lower reporting disproportionality was observed for breast cancer-related reports among VK3-related reports (ROR, 0.183; 95% CI, 0.053–0.631; PRR, 0.184, *p* = 0.0003). Under the assumption of independence, 13.59 co-reports were expected. Although the small observed count warrants caution in interpretation, it represents a marked downward deviation from the expected count and was therefore not excluded solely because the observed count was below three. In the INDI-based sensitivity analysis, 1 co-report was observed compared with the expected 6.00. However, the 95% CI included 1 (ROR, 0.248; 95% CI, 0.050–1.232); therefore, the VK3-specific sensitivity estimate did not meet the predefined criteria, despite a statistically significant Fisher’s exact *p* = 0.0367.

#### 2.1.4. Relationship Between VK4-Related Reports and Breast Cancer-Related Reports

The number of reports related to VK4 reached 155, including 1 breast cancer-related report. Under independence, 1.35 co-reports were expected. No significant reporting disproportionality was observed for breast cancer-related reports among VK4-related reports (ROR, 1.108; 95% CI, 0.222–5.531; PRR, 1.107). In the INDI-based sensitivity analysis, no co-reports were observed compared with the expected 0.53 under the assumption of independence. The corresponding 95% CI was wide and included 1, indicating substantial statistical uncertainty. These data are insufficient to support the use of VK4 as an internal negative control or to draw conclusions regarding a potential structural difference between quinone and hydroquinone forms.

### 2.2. Comparison of Quinone-Type VK with Other Quinone Compounds

**Figure 3:**
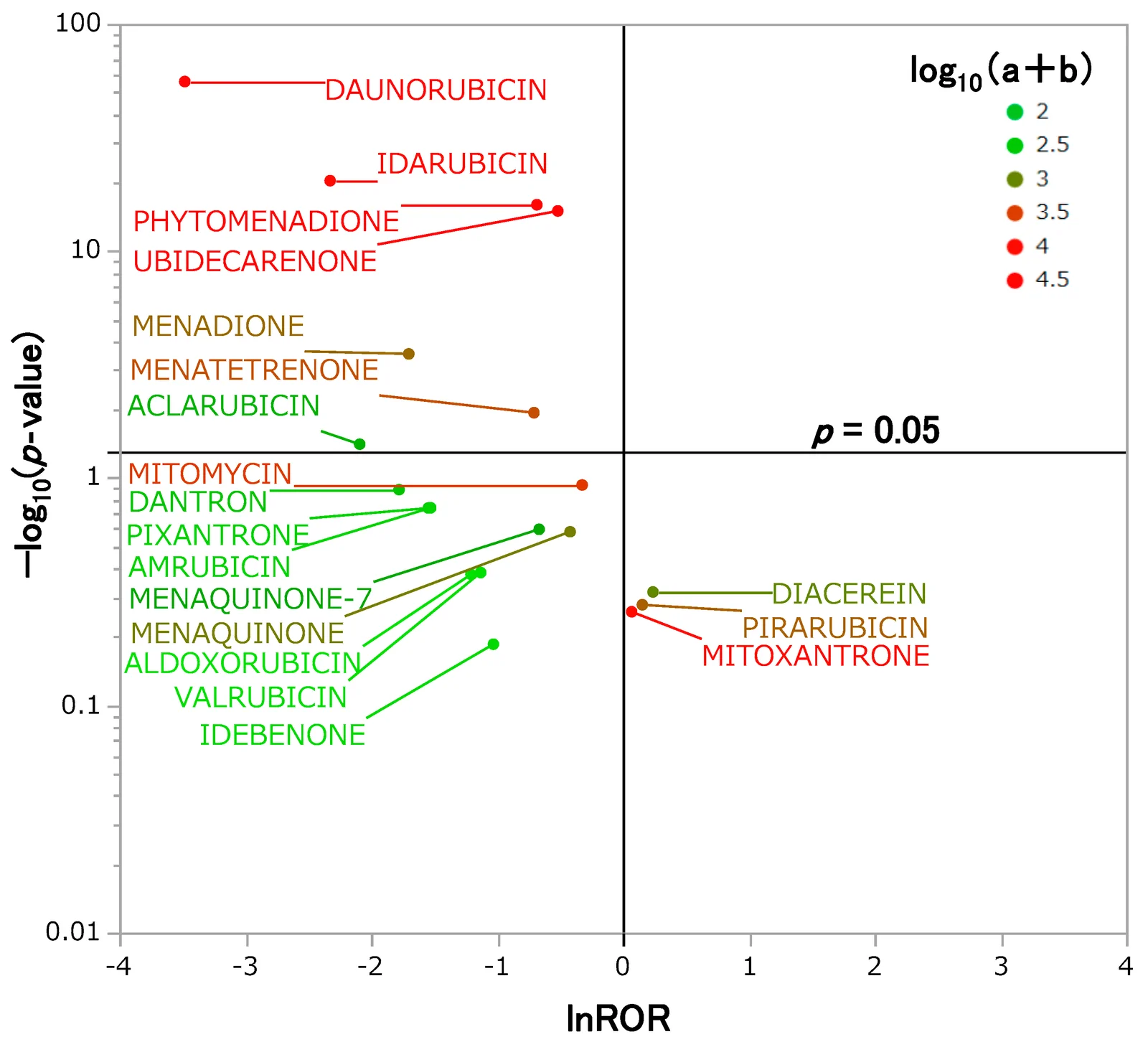
Volcano plot of reporting disproportionality for breast cancer-related reports among 19 of the 34 quinone-containing compounds analyzed in FAERS. The x-axis presents lnROR, and the y-axis presents −log_10_(*p*-value). Color indicates the logarithm of the total number of reports, i.e., log_10_ (a + b). The horizontal line indicates *p* = 0.05.

We compared 5 quinone-type VK homologs with 29 other quinone-containing compounds for reporting disproportionality in breast cancer-related reports, and a subset of the results was visualized using a volcano plot. Among the 34 analyzed compounds, 19 met the prespecified visualization criterion of at least 100 total reports (a + b ≥ 100) and are included in Figure 3. Compounds exhibiting relatively low reporting disproportionality for breast cancer-related reports included VK1, MK-4, and VK3, as well as daunorubicin (ROR, 0.031; 95% CI, 0.012–0.078; PRR, 0.031; *p* < 0.0001), idarubicin (ROR, 0.098; 95% CI, 0.045–0.211; PRR, 0.099; *p* < 0.0001), and ubidecarenone (CoQ10; ROR, 0.597; 95% CI, 0.520–0.685; PRR, 0.599; *p* < 0.0001; Figure 3). For VK3, 2 co-reports were observed versus the expected 13.59. In the volcano plot, anthracycline agents used mainly to treat hematologic malignancies (daunorubicin and idarubicin) were located in the upper-left region, indicating that breast cancer-related reports were relatively sparse. Aclarubicin had 0 observed versus 4.01 expected co-reports with a Fisher’s exact test *p*-value of 0.0389; however, its 95% CI included 1 (ROR, 0.124; 95% CI, 0.008–1.979), and it therefore did not meet the combined predefined criteria. Moreover, not all quinone-containing compounds showed lower reporting disproportionality for breast cancer-related reports. The complete results for all 34 compounds—including observed and expected co-report counts, contingency table cell counts (a–d), RORs, 95% CIs, PRRs, and Fisher’s exact test *p* values—are provided in Supplementary Table S4. The markedly low RORs observed for the hematologic anticancer agents daunorubicin (ROR, 0.031) and idarubicin (ROR, 0.098) are attributable to their therapeutic indications (leukemia rather than breast cancer) and are therefore presented as examples of confounding by indication rather than as evidence supporting a shared quinone-based mechanism. Because this comparison was exploratory and the volcano plot was restricted to compounds with at least 100 total reports (a + b ≥ 100), the *p* values were interpreted as descriptive measures rather than confirmatory hypothesis tests, and no formal adjustment for multiple comparisons was applied.

## 3. Discussion

The principal finding was that breast cancer-related terms were relatively infrequently co-reported with VK-related reports in FAERS (overall ROR, 0.486). In the primary analysis, the predefined criteria for lower reporting disproportionality were also met for VK1, MK-4, VK3, and the integrated VK2 category. For VK3, 2 co-reports were observed versus the expected 13.59, and the upper 95% CI was <1. This finding illustrates why a small observed count should be interpreted in relation to the expected count and the associated statistical uncertainty rather than based on an observed count cutoff alone. In the INDI-based sensitivity analysis, the lower reporting disproportionality was retained for overall VK-related reports and VK1, remained borderline for the integrated VK2 category, and was not retained for MK-4 or VK3. Expected counts are the information contained in the marginal totals but do not account for confounding or reporting bias.

Using FAERS, we compared breast cancer-related co-reporting patterns across VK homologs and other quinone-containing compounds. This cross-compound analysis summarizes differences in reporting distributions but cannot establish the effect of chemical structure on clinical outcomes. As FAERS is a spontaneous reporting database, ROR < 1 in this study only indicated that the reporting frequency of breast cancer-related PTs was relatively low among VK-related reports. It did not demonstrate antitumor efficacy or a reduction in breast cancer risk. In addition, factors such as indications, concomitant medications, underlying diseases, cancer treatment history, supplement use, and reporter characteristics are not adequately controlled in this database, and reporting style or confounding by indication might have affected the results. Therefore, the reporting distributions observed in this study were organized as explanatory hypotheses while being compared with findings from existing basic and epidemiological research.

The compared compounds have markedly different clinical use profiles. VK1 is primarily used to reverse anticoagulant effects and prevent neonatal bleeding due to VK deficiency. MK-4 is used in Japan primarily for postmenopausal osteoporosis. VK3 has limited clinical use in humans; daunorubicin and idarubicin are used mainly for hematologic malignancies; and CoQ10 is used as an adjunct for heart failure and a dietary supplement. Therefore, their underlying reporting populations differ in terms of age, sex, medical-care pathways, observation opportunities, and contact with breast cancer screening. The very low breast cancer-related reporting for leukemia-directed daunorubicin and idarubicin demonstrates that indication structure can dominate the observed pattern. Accordingly, concordant RORs below 1 across several compounds cannot be attributed to a shared quinone mechanism.

We also examined differences among VK homologs. In the primary analysis, the predefined criteria for lower reporting disproportionality were met for VK1, MK-4, VK3, and the integrated VK2 category. For VK3, the 2 observed co-reports were compared with 13.59 expected co-reports, and the corresponding 95% CI was below 1. In contrast, VK4 had 1 observed versus 1.35 expected co-reports, with a wide 95% CI that included 1; therefore, VK4 cannot be considered a suitable internal negative control. These homolog-level findings do not establish a redox- or quinone-structure-dependent effect. Confirmation requires more rigorous control of indication, reporter characteristics, concomitant medications, and suspect-drug role as well as data sources containing sufficient VK4 exposure to support a more informative comparison.

### 3.1. Relationships Between VK-Related Reports and Breast Cancer-Related Reports

#### 3.1.1. VK1

VK1-related reports showed lower reporting disproportionality for breast cancer-related reports, and this pattern remained in the INDI-based sensitivity analysis. Existing evidence on the relationship between VK1 and cancer is inconclusive. Some in vitro and epidemiological studies have suggested antiproliferative effects or inverse associations [22,23,24], whereas studies on triple-negative breast cancer cells have reported pro-proliferative, stem-cell-like effects that differ from those of MK-4 [25]. VK1 can also be converted to MK-4 via UBIAD1 in vivo [26,27]. These findings provide biological context but do not resolve the FAERS reporting pattern.

#### 3.1.2. MK-4

MK-4-related reports showed lower reporting disproportionality in the primary analysis; however, the MK-4-specific estimate was no longer statistically significant in the INDI-based sensitivity analysis. The integrated VK2 category retained a borderline lower reporting disproportionality; however, this pooled estimate does not provide a similar finding for each individual homolog. In nonclinical models, MK-4 has been reported to induce ROS-associated apoptosis and, in triple-negative breast cancer cells, nonapoptotic cell death accompanied by autophagy [8,9,10,28,29,30,31,32,33]. Additional MK-4-associated cellular responses and molecular mechanisms have been described, including Bak modification [32], monocytic differentiation [34], and NF-κB modulation [35,36]. These findings provide mechanistic context but do not explain the FAERS co-reporting pattern.

#### 3.1.3. VK3

VK3-related reports met the predefined criteria for lower reporting disproportionality in the primary analysis (2 observed versus 13.59 expected co-reports; ROR = 0.183, 95% CI, 0.053–0.631). In the INDI-based sensitivity analysis, 1 co-report was observed versus 6.00 expected; however, the 95% CI included 1. Therefore, the VK3-specific sensitivity estimate did not meet the predefined criteria.

VK3 has a strong capacity to generate ROS through one-electron reduction and redox cycling [5,6,37] and has demonstrated ROS-dependent cytotoxicity in breast cancer cell lines [20,21]. It also causes nonselective toxicity in normal cells, limiting its clinical use [5,6,38]. This restricted use likely influences the composition of VK3-related FAERS reports.

#### 3.1.4. VK4

VK4 did not meet the predefined criteria for lower reporting disproportionality in either examination. In the primary examination, 1 co-report was detected versus 1.35 expected, and the corresponding 95% CI was wide and included 1. Therefore, the available VK4 data do not support the inference of a structure- or redox-dependent difference among VK homologs.

### 3.2. Comparative Examination of Quinone-Type VK and Other Quinone Compounds

Among the 34 quinone-containing compounds, 6 met the predefined criteria for lower breast cancer-related reporting disproportionality: VK1, MK-4, VK3, daunorubicin, idarubicin, and CoQ10. These compounds were compared based on their pharmacological backgrounds and clinical use contexts. The low reporting disproportionality observed for daunorubicin and idarubicin likely reflects their leukemia-directed indications, reflecting the potential influence of confounding by indication rather than providing evidence for a shared quinone-based mechanism.

#### 3.2.1. Anthracycline Quinone Compounds

Daunorubicin and idarubicin are anthracycline anticancer agents with quinone structures. In leukemia cell lines, these agents have been associated with apoptosis and autophagy, although the contribution of ROS may differ between agents [39,40,41,42]. In the present study, a low breast cancer-related reporting disproportionality for these leukemia-directed agents likely reflects their therapeutic indications, potentially contributing to the observed reporting pattern and reflecting the influence of confounding by indication rather than supporting a quinone-structure-based explanation.

#### 3.2.2. CoQ10

CoQ10, similar to VK homologs and anthracycline agents, possesses a quinone structure; however, its physiological role differs substantially. Specifically, CoQ10 differs from other quinone compounds in that direct cell death induction is not its main action. CoQ10 functions primarily as a cytoprotective redox molecule involved in the suppression of lipid peroxidation and ferroptosis through the FSP1-CoQ10H2 pathway, contributing to cell membrane stabilization and mitochondrial function maintenance [43,44]. Therefore, CoQ10 is generally considered a cytoprotective redox-buffering molecule. In breast cancer models, CoQ10 and UBIAD1 have also been reported to alter plasma membrane mechanical properties, disrupt extracellular matrix (ECM)-mediated oncogenic signaling, and reduce resistance to ferroptosis [45].

CoQ10-related reporting may be influenced by its clinical use context, including its use as a supportive or adjunctive agent. Preclinical studies summarized in a scoping review have examined CoQ10 for the mitigation of doxorubicin-associated cardiotoxicity, particularly in relation to anthracycline-induced oxidative injury [46]. Although CoQ10 is a quinone-containing compound, its mode of action differs markedly from that of quinone-type VK homologs. Clinically used quinone-type VK homologs, particularly MK-4, can be incorporated into two-electron reduction pathways under physiological conditions, whereas under conditions of disrupted redox homeostasis, they might diverge into cell death pathways through redox reactions or ROS signaling. By contrast, CoQ10 is clearly distinguished by its primary function as a cytoprotective redox-buffering molecule based on the suppression of lipid peroxidation.

Therefore, even among compounds sharing the same quinone structure, the implications of the relatively low reporting disproportionality observed in FAERS are not necessarily identical between compounds that can drive ROS production and contribute to cell death pathways (e.g., some anthracycline agents, VK3) and compounds that function mainly in a cytoprotective manner through mechanisms such as lipid peroxidation suppression (e.g., CoQ10). The relatively low reporting disproportionality observed in this study may reflect heterogeneous reporting structures and clinical use contexts across quinone-containing compounds and should not be interpreted as evidence of a common biological effect attributable to the quinone structure.

### 3.3. Clinical Significance of Quinone-Type VK Homologs

Compared with other compounds possessing the same quinone structure, VK homologs are distinctive in that they can participate in two-electron reduction pathways under physiological conditions [1,2,3] unlike anthracycline agents, which can induce ROS production through redox reactions, or CoQ10, which functions mainly in a cytoprotective manner. These differences provide a biological background for considering VK homologs but should not be interpreted as explanations for the relatively low reporting disproportionality for breast cancer-related reports observed for quinone-type VK-related reports in this study.

### 3.4. Limitations

This study had several limitations. First, as a spontaneous reporting database, FAERS includes inherent reporting biases such as underreporting, selective reporting, and effects of reporting media. Therefore, the ROR and PRR data calculated in this study do not directly reflect incidence or risk in the relevant populations; instead, they are signal indices based on the reporting structure.

Second, FAERS does not provide sufficient clinical information on dose, the duration of treatment, treatment adherence, indications, concomitant medications, underlying diseases, or disease severity. Therefore, it is difficult to fully adjust for confounding by indication or the effects of concomitant drugs. In addition, some reports included multiple VK homologs, making it impossible to completely separate the effects among homologs. Because the temporal relationship between administration and breast cancer onset cannot be rigorously verified, causality cannot be inferred. Even when multiple drug names are recorded in a single FAERS report, this does not necessarily reflect actual concomitant use or administration relationships. Thus, the inclusion of multiple VK homologs in some reports does not necessarily imply combination therapy and may instead reflect duplicate entries, reporting practices, or other features of spontaneous reporting data. In particular, breast cancer-related reports in FAERS are enriched for oncology, endocrine therapy, targeted therapy, and bone-modifying agents. Thus, VK-related reports may show an ROR of <1 partly because they are compared with a background reporting population that is enriched for cancer-related reporting. This comparator imbalance was not controlled in the present design and cannot be distinguished from a potential compound-related effect. In addition, breast cancer-related MedDRA terms in FAERS do not necessarily represent drug-induced adverse events. For malignancies, such terms reflect the underlying disease, therapeutic indication, medical history, or treatment context. Because the present analysis identified breast cancer-related reports solely from the REAC table, it could not distinguish breast cancer terms reported as adverse events from those recorded as indications or clinical background. To address this limitation, we performed an INDI-based sensitivity analysis that excluded reports containing breast cancer-related indication terms recorded in the INDI table; the results are provided in Supplementary Table S5. Notably, this INDI-based exclusion removed approximately 53% of VK-related reports (from 32,156 to 15,178), compared with a 12% reduction in the total database, indicating that breast cancer-related indication terms were disproportionately recorded among VK-related reports. Because the exposure group and the comparator background were reduced asymmetrically, the lower ROR observed after exclusion (e.g., from 0.486 to 0.348 for overall VK-related reports) should be interpreted only as directional consistency and not as quantitative evidence that confounding was eliminated. The changes in the point estimate may partly reflect this reconstruction of the analysis population and its denominators. Furthermore, this exclusion addresses only breast cancer terms recorded as indications and does not control for the broader enrichment of the comparator background in cancer-related reporting; therefore, residual confounding by indication remains possible. Moreover, indication information in FAERS is frequently missing or incompletely recorded. Because reports lacking recorded breast cancer indications were retained in the analysis, some reports with unrecorded breast cancer indications may have remained in the sensitivity analysis dataset. Accordingly, the INDI-based analysis cannot be considered to have eliminated confounding by indication and should be interpreted as a supplementary analysis.

Third, although breast cancer outcomes were defined using Standardized MedDRA Queries (SMQs; narrow scope), detailed clinical information, such as stage, molecular subtype, and diagnostic certainty, could not be obtained. Estimates based on small observed cell counts can be sensitive to the 0.5 continuity correction. Therefore, expected co-report counts were reported to help distinguish a small observed count from a small expected count; however, the expected counts are descriptive and do not correct reporting bias or confounding. Statistical classification required an ROR of <1, an upper 95% confidence limit below 1, and Fisher’s exact test *p* < 0.05. Because multiple components and analytical comparisons were evaluated, the potential for false-positive findings remains. The criterion of a + b ≥ 100 was used solely to determine the compounds that were displayed in the volcano plot, and *p* values from the cross-compound comparisons were interpreted as descriptive rather than confirmatory.

## 4. Materials and Methods

### 4.1. Data Source and Construction of the Integrated Table

Analyses were conducted using public quarterly data from the FAERS database [47], which reports seven tables: DEMO, DRUG, REAC, OUTC, RPSR, INDI, and THER. This study used data from the first quarter of 2004 through the third quarter of 2024. The DRUG and REAC tables were merged by PRIMARYID to construct an integrated table for analysis. To avoid overestimation in report counts, an integrated table was constructed and duplicate records were removed in line with previous FAERS analyses [48,49]. The DRUG and REAC tables were deduplicated at the record level before integration. Although the removal of duplicate records reduced the number of records in both the DRUG and REAC tables, the number of unique PRIMARYIDs remained unchanged before and after deduplication: 18,321,080 in the DRUG table and 18,328,666 in the REAC table. The deduplicated DRUG and REAC tables were then integrated using an inner join on PRIMARYID. The resulting analytical dataset contained 18,320,966 unique PRIMARYIDs, corresponding to unique FAERS reports. This number was defined as the total number of analyzed FAERS reports.

### 4.2. Extraction of Quinone- and Hydroquinone-Containing Compounds

For drug names listed in the FAERS DRUG table, the PubChem database was searched using the Python library PubChemPy (with Python version 3.9.23), and Simplified Molecular Input Line Entry System (SMILES) strings corresponding to each drug were obtained [50,51]. The obtained SMILES strings were imported into Molecular Operating Environment version 2022.02 (Chemical Computing Group, Montreal, Quebec, Canada), and substructure searches were performed using SMiles ARbitrary Target Specification (SMARTS) expressions [48,49,52].

Because SMILES representations of quinone structures vary depending on the treatment of aromaticity, multiple SMARTS expressions were used. Specifically, SMARTS expressions capable of detecting para-benzoquinone, para-benzoquinone-singleA, and para-benzoquinone-doubleA were used to extract quinone skeletons. To identify hydroquinone-type homologs, SMARTS expressions for hydroquinone were also used. Components matching any of these SMARTS expressions were extracted as candidate components, and generic names, structural formulas, alternative names, and duplicates were individually reviewed to determine the final components for analysis. The SMARTS expressions are listed in Supplementary Table S1.

The VK homologs analyzed in this study included VK1, MK-n, MK-4, MK-7, VK3, and VK4. In the exploratory compound comparison, in addition to the quinone-type VK homologs, 29 other components containing quinone skeletons were analyzed after *a priori* exclusion of doxorubicin and epirubicin, which are used as breast cancer therapies. These 34 agents were analyzed using the same reporting disproportionality method. The 34 quinone-containing compounds extracted using these SMARTS queries are listed in Supplementary Table S2. VK4 was included in the homolog-specific analyses because it is a VK homolog, but it was excluded from the exploratory comparison among quinone-type compounds because it is a hydroquinone-type compound. Doxorubicin and epirubicin were excluded a priori because breast cancer is an approved indication for both drugs, which would, by definition, enrich their reports with breast cancer-related terms. In contrast, the leukemia-directed anthracyclines retained in the comparison would be expected to show low reporting of breast cancer-related terms for the same indication-driven reason and are therefore interpreted as examples of confounding by indication rather than as supportive evidence. Contingency table cell counts (a–d), RORs, 95% CIs, PRRs, and *p* values of Fisher’s exact test for all 34 compounds are provided in Supplementary Table S4.

### 4.3. Definition of VK-Related and Breast Cancer-Related Reports

The unit of analysis in this study was the FAERS report unit PRIMARYID. When one or more names of a given component appeared within a PRIMARYID, the report was defined as related to that component. Therefore, this study did not distinguish between monotherapy and combination therapy. When multiple VK homologs or other drugs were listed in the same report, each component was dichotomized and analyzed separately. VK-related reports were defined as reports listing VK1, MK-n, MK-4, MK-7, VK3, and/or VK4. Homolog-specific analyses were then conducted for VK1-, MK-n–, MK-4–, MK-7–, VK3-, and VK4-related reports. All drug records were included irrespective of their reported role codes, including primary suspect, secondary suspect, concomitant, and interacting drugs. Therefore, the presence of a drug name in a report indicates only that the drug was recorded in the same FAERS report; it does not imply that the drug caused or was suspected of causing the breast cancer-related adverse events represented by those terms.

Breast cancer-related reports were defined using all valid Preferred Terms (PTs) included in the narrow scope of the lower-level SMQs “Breast malignant tumours” (20000198) and “Breast tumours of unspecified malignancy” (20000199), which belong to the SMQ “Breast neoplasms, malignant and unspecified” (20000149) [53,54]. The combined narrow-scope SMQ lists contained 66 PT entries, corresponding to 65 unique PTs (Supplementary Table S3). Breast cancer-related reports were identified by exact PT matching to the unique PTs in this predefined combined list. Even when multiple such PTs were included within a single PRIMARYID, the report was counted as a breast cancer-related report only once. Because these PTs were identified from the REAC table, they are treated as reported terms rather than as confirmed drug-induced adverse events. In the context of malignancies, such terms may also reflect the underlying disease, therapeutic indication, medical history, or broader clinical content (see Section 3.4).

### 4.4. Disproportionality Analysis

**Figure 4:**
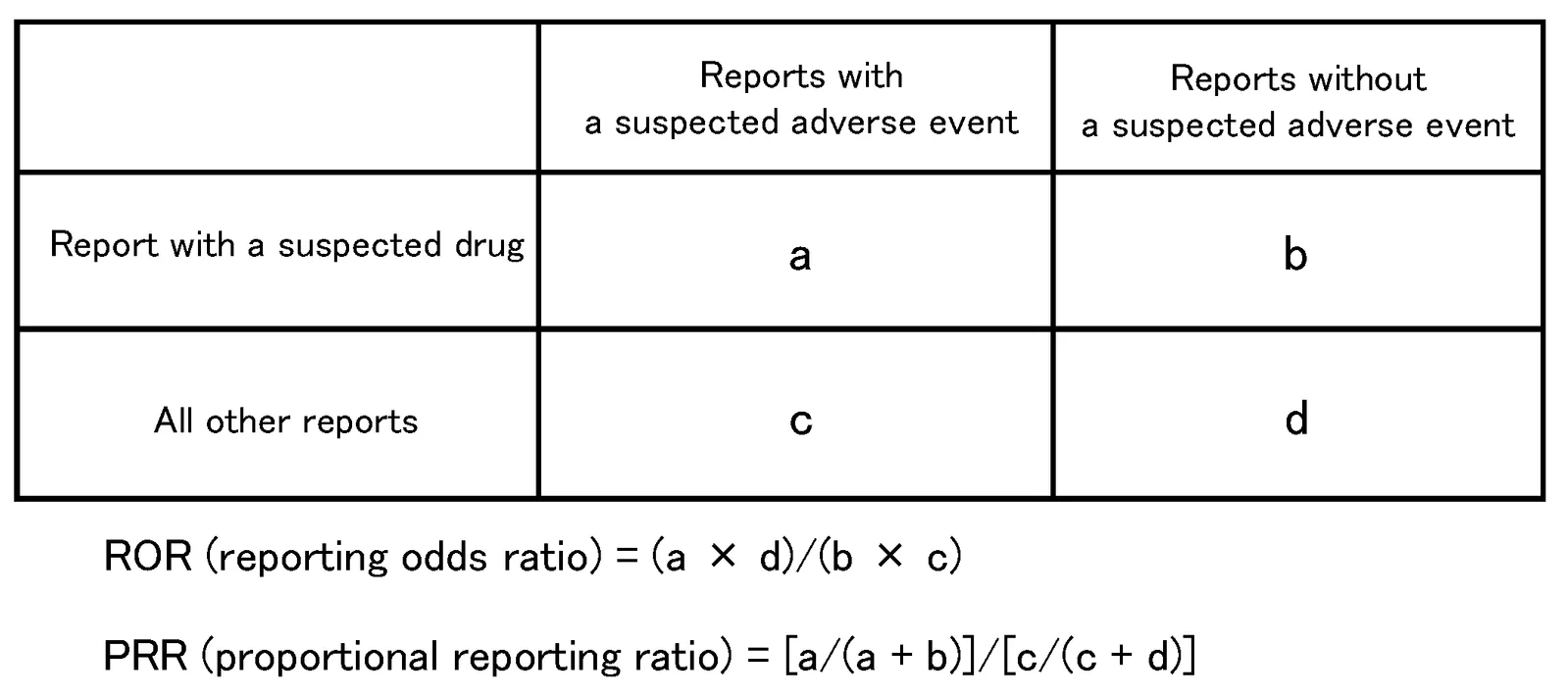
Cross-tabulation and formula used to calculate the ROR and PRR for a reporting term of interest. The table is organized with reports for the suspected drug, all other reports, reports with the reporting term, and reports without the reporting term (a–d represent the number of reports).

For each component analyzed, a 2 × 2 contingency table was constructed according to the presence or absence of breast cancer-related reports and the presence or absence of reports related to the component. Fisher’s exact test was performed, and the ROR with its 95% CI and the PRR were calculated. ROR was calculated as (a × d)/(b × c), and PRR was calculated as [a/(a + b)]/[c/(c + d)] (Figure 4). For all 2 × 2 tables, a Haldane–Anscombe continuity correction of 0.5 was added to every cell to calculate the ROR, PRR, and 95% CI [55,56], whereas the *p* values of Fisher’s exact test were calculated using the original integer counts. The expected number of breast cancer-related co-reports under the assumption of independence was calculated from the uncorrected marginal totals as E = (a + b) (a + c)/N, where N = a + b + c + d.

Lower reporting disproportionality was defined as an ROR of <1, an upper 95% confidence limit of <1, and Fisher’s exact test *p* < 0.05. No additional cutoff based solely on the observed co-report count (a) was imposed because a small observed count is the quantity being evaluated in an inverse-disproportionality analysis. The expected count and CI were reported to provide context and characterize statistical uncertainty. The PRR was used as an auxiliary measure of directional consistency. A volcano plot was generated for all 34 components, with ln(ROR) on the x-axis and −log_10_(*p*-value) on the y-axis [57]. The volcano plot was generated using JMP Student Edition version 19.1.3 (JMP Statistical Discovery LLC, Cary, NC, USA). The prespecified visualization criterion was at least 100 total reports (a + b ≥ 100), along with computable ROR and *p* values from the Fisher’s exact test; 19 compounds met this criterion. Complete results for all 34 compounds are provided in Supplementary Table S4. Because multiple compounds were evaluated, *p* values were interpreted as descriptive indexes, and no formal multiplicity adjustment for multiple comparisons was applied. The INDI-based sensitivity analysis used the same set of narrowly defined breast cancer-related PTs to identify indication records. PRIMARYIDs with a matching INDI term were excluded before the reintegration of the remaining DRUG and REAC records. RORs, PRRs, 95% CIs, *p* values of the Fisher’s exact test, and expected co-report counts were then recalculated using the same framework.

**Figure 5:**
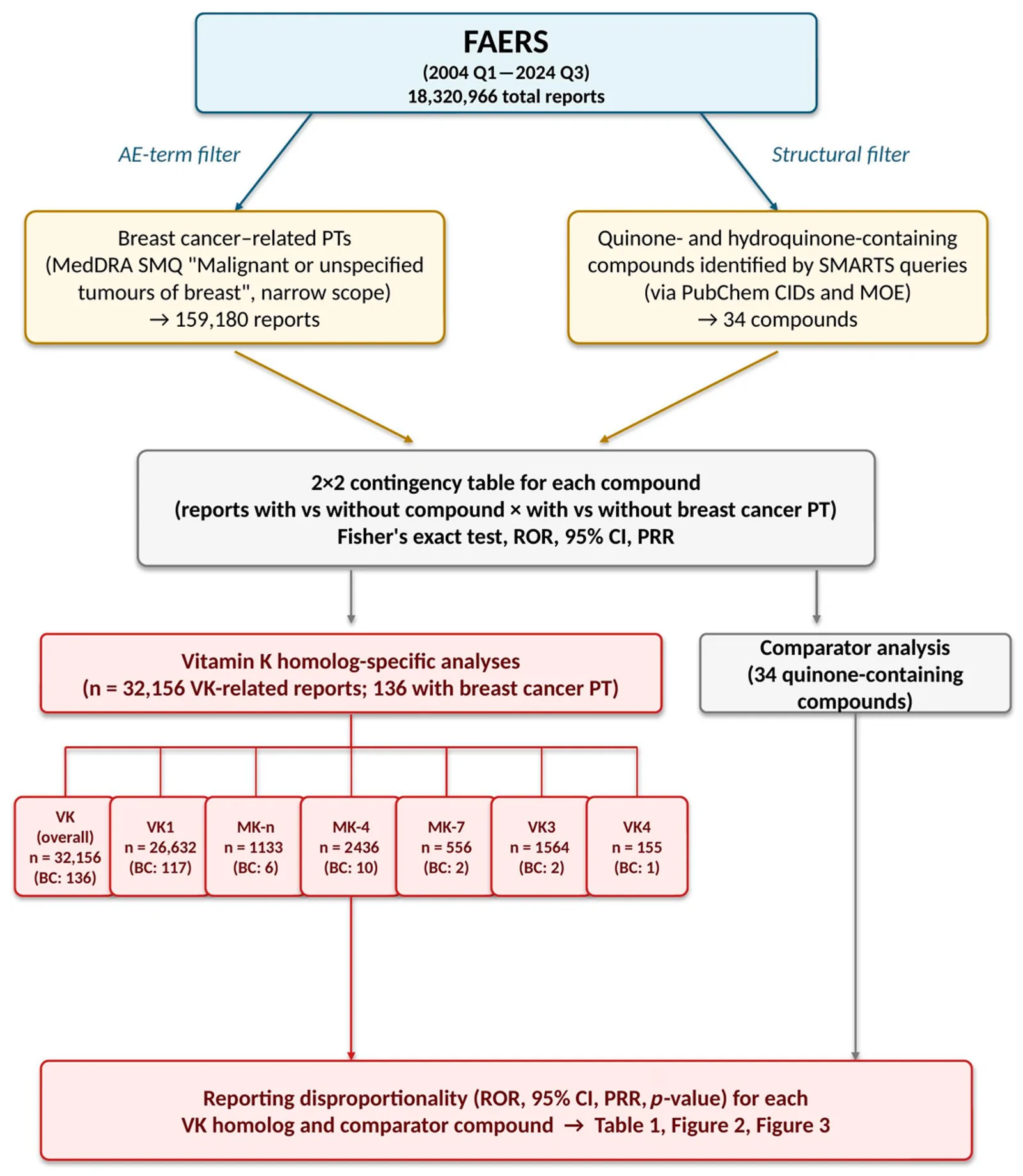
Study flow diagram of the FAERS-based pharmacovigilance analysis of VK homologs and breast cancer-related reports. Reports in the FAERS database (2004 Q1–2024 Q3; 18,320,966 total reports) were filtered through two parallel pipelines: a reporting-term filter that identified breast cancer-related reports using narrow-scope PTs from the lower-level SMQs “Breast malignant tumours” and “Breast tumours of unspecified malignancy,” which belong to the SMQ “Breast neoplasms, malignant and unspecified” (159,180 reports), and a structural filter that used SMARTS substructure searches against PubChem-derived SMILES in Molecular Operating Environment to identify quinone- and hydroquinone-containing compounds, from which 34 quinone-containing compounds were selected for the comparator analysis. For each compound, a 2 × 2 contingency table was constructed, and the ROR and its associated 95% CI, PRR, and the *p*-value of the Fisher’s exact test were calculated. VK homolog-specific analyses (*n* = 32,156 VK-related reports, of which 136 were breast cancer-related) examined all VK homologs and the six homologs separately (VK1, MK-n, MK-4, MK-7, VK3, and VK4). The comparator analysis examined all 34 quinone-containing compounds. Note that VK4 (the hydroquinone-type homolog) was included in the homolog-level analyses but excluded from the comparator volcano plot (Figure 3), which was restricted to quinone-type compounds. BC, breast cancer.

The overall analytical flow from data extraction to final reporting disproportionality analysis is presented in Figure 5. From the total FAERS reports, data extracted through two parallel pathways, a reporting-term filter (MedDRA SMQ) and a compound-structure filter (SMARTS), were integrated. For each component, reporting disproportionality analysis was performed using a 2 × 2 contingency table, and results were derived in two analytical streams: VK homolog-specific analyses and comparative analyses with 34 quinone-containing compounds.

## 5. Conclusions

This study found that breast cancer-related terms were relatively infrequently co-reported with VK-related reports in FAERS. In the primary analysis, the predefined criteria for lower reporting disproportionality were met for VK1, MK-4, VK3, and the integrated VK2 category. For VK3, 2 co-reports were observed and 13.59 were expected. Because the VK4 estimate relied on 1 observed versus 1.35 expected co-reports and had a wide 95% CI that included 1, the differences between homologs cannot be interpreted as evidence of a redox- or quinone-structure-dependent effect. In the INDI-based sensitivity analysis, the lower reporting disproportionality was retained for overall VK-related reports and VK1, remained borderline for the integrated VK2 category, and was not retained for MK-4 or VK3. These findings are hypothesis-generating, and they do not directly demonstrate causality, reduced breast cancer risk, or clinical efficacy. The results may indicate the influence of indications, patient background, concomitant drugs, use scenarios, reporting structure, the indication structure of the comparator reporting population, and other factors. Future validation through mechanistic studies and analytical epidemiological studies with more rigorous control of potential confounding—including reporter characteristics, concomitant medications, indication structures, and suspect-drug role—is warranted to clarify the biological and clinical relevance of the observed reporting patterns.

## Figures and Tables

**Table 1:** Observed and expected co-report counts, reporting odds ratios (RORs), and proportional reporting ratios (PRRs) for breast cancer-related reports across VK homologs in FAERS.

VK Type	a	b	c	d	Expected E	ROR (95% CI)	PRR	*p*-Value
VK	136	32,020	159,044	18,129,766	279.38	0.486 (0.411–0.575)	0.488	<0.0001 *
VK1	117	26,515	159,063	18,135,271	231.39	0.505 (0.421–0.606)	0.507	<0.0001 *
VK2	18	4088	159,162	18,157,698	35.67	0.516 (0.327–0.815)	0.518	0.0018 *
MK-n	6	1127	159,174	18,160,659	9.84	0.658 (0.304–1.422)	0.660	0.2626
MK-4	10	2426	159,170	18,159,360	21.16	0.494 (0.269–0.905)	0.496	0.0115 *
MK-7	2	554	159,178	18,161,232	4.83	0.514 (0.148–1.782)	0.517	0.2539
VK3	2	1562	159,178	18,160,224	13.59	0.183 (0.053–0.631)	0.184	0.0003 *
VK4	1	154	159,179	18,161,632	1.35	1.108 (0.222–5.531)	1.107	1.0000

Total number of reports, N = 18,320,966; total number of breast cancer-related reports = 159,180. E is the expected number of co-reports under independence, calculated as E = (a + b) (a + c)/N from the uncorrected marginal totals. CI, confidence interval. * Fisher’s exact *p* < 0.05. A lower reporting disproportionality was defined as ROR of <1, an upper 95% confidence limit of <1, and Fisher’s exact *p* < 0.05.

## Data Availability

The data analyzed in this study are publicly available from the FDA Adverse Event Reporting System.

## Supplementary Materials

The following supporting information can be downloaded at: https://www.mdpi.com/article/10.3390/ph19081181/s1, Table S1: SMARTS expressions; Table S2: The 34 quinone-containing compounds included in the analysis; Table S3: Breast cancer-related PTs included in the two lower-level Standardized MedDRA Queries; Table S4: Observed and expected co-report counts, contingency cell counts (a–d), ROR, 95% CI, PRRs, and Fisher’s exact *p* values for all 34 quinone-containing compounds; Table S5: INDI-based sensitivity analysis of reporting disproportionality for breast cancer-related reports, including observed and expected co-report counts, after excluding reports with breast cancer-related indication terms documented in the INDI table.

## Abbreviations

The following abbreviations are used in this manuscript:

PRR	Proportional reporting ratio
PT	Preferred Terms
ROR	Reporting odds ratio
ROS	Reactive oxygen species
SMARTS	SMiles ARbitrary Target Specification
SMILES	Simplified Molecular Input Line Entry System
