# Supplementary material for: Disproportionality Analysis of Breast Cancer-Related Reports Across Vitamin K Homologs in the FDA Adverse Event Reporting System
Source: Pharmaceuticals (Basel). 2026 Jul 28;19(8):1181. doi: 10.3390/ph19081181 (PMC13516650; doi:10.3390/ph19081181)
Supplement: Supplementary file 1 [file pharmaceuticals-19-01181-s001.zip › pharmaceuticals-4416938-supplementary.pdf]

Supplementary Table S1

SMILES ARbitrary Target Specification (SMARTS) queries used for the identification of quinone- and hydroquinone-containing compounds.

p-Benzoquinone (para-benzoquinone)

[#6]-1(-[#6]=[#6]-[#6](-[#6]=[#6]-1)=[#8])=[#8]

para-benzoquinone-singleA

[#6]-1(-[#6]:[#6]-[#6](-[#6]=[#6]-1)=[#8])=[#8]

para-benzoquinone-doubleA

[#6]-1(-[#6]:[#6]-[#6](-[#6]:[#6]-1)=[#8])=[#8]

hydroquinone (explicit H)

c1(ccc(cc1)-[#8]-[#1])-[#8]-[#1]

hydroquinone (implicit H)

c1(ccc(cc1)-[#8])-[#8]

Supplementary Table S2

List of the 34 quinone-containing compounds extracted using SMARTS queries and included in the exploratory comparison analysis.

Aclarubicin	Mitomycin
Aldoxorubicin	Mitoquinone
Amrubicin	Mitoxantrone
Carboquone	Napabucasin
Chloranil	Phytomenadione
Cochineal	Pirarubicin
Dantron	Pixantrone
Daunorubicin	Quinone
Diacerein	Sabarubicin
Diaziquone	Sepantronium
Frangulin	Seratrodast
Idarubicin	Tanespimycin
Idebenone	Thymoquinone
Menadione	Triaziquone
Menaquinone	Ubidecarenone
Menaquinone-7	Valrubicin
Menatetrenone	Zorubicin

* Doxorubicin and epirubicin were excluded a priori because they are used for breast cancer therapy.

Supplementary Table S3 Preferred Terms (PTs) and MedDRA codes included in the narrow scope of the lower-level SMQs "Breast malignant tumours" (20000198) and "Breast tumours of unspecified malignancy" (20000199), which belong to the MedDRA SMQ "Breast neoplasms, malignant and unspecified" (20000149), Version 27.1.

20000149 Breast neoplasms, malignant and unspecified (SMQ)

20000198 Breast malignant tumours (SMQ)

SMQ scope	Category	Preferred Term	MedDRA code
Narrow	A	Apocrine breast carcinoma	10066206
Narrow	A	Breast ablation	10090883
Narrow	A	Breast cancer	10006187
Narrow	A	Breast cancer female	10057654
Narrow	A	Breast cancer in situ	10006189
Narrow	A	Breast cancer male	10061020
Narrow	A	Breast cancer metastatic	10055113
Narrow	A	Breast cancer recurrent	10006198
Narrow	A	Breast cancer stage I	10006199
Narrow	A	Breast cancer stage II	10006200
Narrow	A	Breast cancer stage III	10006201
Narrow	A	Breast cancer stage IV	10006202
Narrow	A	Breast sarcoma	10068582
Narrow	A	Breast sarcoma metastatic	10068583
Narrow	A	Breast sarcoma recurrent	10068584
Narrow	A	Electron radiation therapy to breast	10014437
Narrow	A	Extended radical mastectomy	10015721
Narrow	A	Gamma radiation therapy to breast	10017677
Narrow	A	HER2 low breast cancer	10089162
Narrow	A	HER2 negative breast cancer	10083232
Narrow	A	HER2 positive breast cancer	10065430
Narrow	A	Hormone receptor negative HER2 positive breast cancer	10085561
Narrow	A	Hormone receptor positive breast cancer	10083234
Narrow	A	Hormone receptor positive HER2 negative breast cancer	10085481
Narrow	A	Hormone refractory breast cancer	10076935
Narrow	A	Inflammatory carcinoma of breast recurrent	10021977
Narrow	A	Inflammatory carcinoma of breast stage III	10021978
Narrow	A	Inflammatory carcinoma of breast stage IV	10021979
Narrow	A	Inflammatory carcinoma of the breast	10021980
Narrow	A	Intraductal papillary breast neoplasm	10073540
Narrow	A	Intraductal proliferative breast lesion	10073094
Narrow	A	Invasive breast carcinoma	10075713
Narrow	A	Invasive ductal breast carcinoma	10073095
Narrow	A	Invasive lobular breast carcinoma	10073096
Narrow	A	Invasive papillary breast carcinoma	10073098
Narrow	A	Lobular breast carcinoma in situ	10073099
Narrow	A	Malignant nipple neoplasm	10062051
Narrow	A	Malignant nipple neoplasm female	10053129
Narrow	A	Malignant nipple neoplasm male	10053128
Narrow	A	Mastectomy	10026878
Narrow	A	Medullary carcinoma of breast	10027095
Narrow	A	Metaplastic breast carcinoma	10073100
Narrow	A	Modified radical mastectomy	10027799
Narrow	A	Mucinous breast carcinoma	10073101
Narrow	A	Neuroendocrine breast tumour	10073103
Narrow	A	Oestrogen receptor assay positive	10054054
Narrow	A	Paget's disease of nipple	10033364
Narrow	A	Photon radiation therapy to breast	10034949
Narrow	A	Postmastectomy lymphoedema syndrome	10036390
Narrow	A	Primary breast lymphoma	10081036
Narrow	A	Progesterone receptor assay positive	10054057
Narrow	A	Radical mastectomy	10037773
Narrow	A	Radiotherapy to breast	10062090
Narrow	A	Secretory breast carcinoma	10088985
Narrow	A	Simple mastectomy	10040700
Narrow	A	Squamous cell breast carcinoma	10079307
Narrow	A	Triple negative breast cancer	10075566
Narrow	A	Triple positive breast cancer	10083233
Narrow	A	Tubular breast carcinoma	10073104
Narrow	A	X-ray therapy to breast	10048199

20000199 Breast tumours of unspecified malignancy (SMQ)

SMQ scope	Category	Preferred Term	MedDRA code
Narrow	A	Adenomyoepithelioma of breast	10088008
Narrow	A	Breast ablation	10090883
Narrow	A	Breast neoplasm	10006279
Narrow	A	Breast tumour excision	10079326
Narrow	A	Nipple neoplasm	10056286
Narrow	A	Phyllodes tumour	10071776

Supplementary Table S4. Full 2 × 2 contingency table cell counts, observed and expected breast cancer-related co-report counts, reporting odds ratios (RORs), 95% confidence intervals, proportional reporting ratios, and Fisher's exact *p* values for the 34 quinone-containing compounds included in the comparison analysis.

Drug	a	b	c	d	Drug Total	Expected E	ROR (95% CI)	PRR	*p*-value	
Daunorubicin	4	16,652	159,176	18,145,134	16,656	144.71	0.031 (0.012 – 0.078)	0.031	<0. 0001	*
Idarubicin	6	7,584	159,174	18,154,202	7,590	65.95	0.098 (0.045 – 0.211)	0.099	<0. 0001	*
VK1 (Phytomenadione)	117	26,515	159,063	18,135,271	26,632	231.39	0.505 (0.421 – 0.606)	0.507	<0. 0001	*
Ubidecarenone	204	39,068	158,976	18,122,718	39,272	341.21	0.597 (0.520 – 0.685)	0.599	<0. 0001	*
VK3 (Menadione)	2	1,562	159,178	18,160,224	1,564	13.59	0.183 (0.053 – 0.631)	0.184	0.0003	*
MK-4 (Menatetrenone)	10	2,426	159,170	18,159,360	2,436	21.16	0.494 (0.269 – 0.905)	0.496	0.0115	*
Aclarubicin	0	461	159,180	18,161,325	461	4.01	0.124 (0.008 – 1.979)	0.125	0.0389	*
Mitomycin	21	3,388	159,159	18,158,398	3,409	29.62	0.724 (0.474 – 1.106)	0.726	0.1166	
Dantron	0	337	159,180	18,161,449	337	2.93	0.169 (0.011 – 2.708)	0.170	0.1288	
Amrubicin	0	262	159,180	18,161,524	262	2.28	0.217 (0.014 – 3.484)	0.219	0.1818	
Pixantrone	0	267	159,180	18,161,519	267	2.32	0.213 (0.013 – 3.419)	0.215	0.1825	
MK-7 (Menaquinone-7)	2	554	159,178	18,161,232	556	4.83	0.514 (0.148 – 1.782)	0.517	0.2539	
MK-n (Menaquinone)	6	1,127	159,174	18,160,659	1,133	9.84	0.658 (0.304 – 1.422)	0.660	0.2626	
Valrubicin	0	176	159,180	18,161,610	176	1.53	0.323 (0.020 – 5.188)	0.325	0.4133	
Aldoxorubicin	0	190	159,180	18,161,596	190	1.65	0.299 (0.019 – 4.805)	0.301	0.4200	
Diacerein	10	942	159,170	18,160,844	952	8.27	1.271 (0.692 – 2.335)	1.268	0.4836	
Pirarubicin	18	1,807	159,162	18,159,979	1,825	15.86	1.168 (0.739 – 1.846)	1.166	0.5285	
Mitoxantrone	76	8,133	159,104	18,153,653	8,209	71.32	1.073 (0.857 – 1.344)	1.072	0.5520	
Idebenone	0	159	159,180	18,161,627	159	1.38	0.358 (0.022 – 5.743)	0.360	0.6520	
Napabucasin	0	99	159,180	18,161,687	99	0.86	0.573 (0.036 – 9.231)	0.575	1.0000	
Tanespimycin	0	34	159,180	18,161,752	34	0.30	1.654 (0.101 – 26.972)	1.644	1.0000	
Seratrodast	0	19	159,180	18,161,767	19	0.17	2.926 (0.177 – 48.456)	2.877	1.0000	
Frangulin	0	14	159,180	18,161,772	14	0.12	3.934 (0.235 – 65.956)	3.837	1.0000	
Quinone	0	13	159,180	18,161,773	13	0.11	4.226 (0.251 – 71.089)	4.111	1.0000	
Sepantronium	0	12	159,180	18,161,774	12	0.10	4.564 (0.270 – 77.086)	4.427	1.0000	
Cochineal	0	10	159,180	18,161,776	10	0.09	5.433 (0.318 – 92.722)	5.232	1.0000	
Zorubicin	0	7	159,180	18,161,779	7	0.06	7.606 (0.434 – 133.187)	7.193	1.0000	
Sabarubicin	0	4	159,180	18,161,782	4	0.03	12.677 (0.683 – 235.476)	11.510	1.0000	
Carboquone	0	2	159,180	18,161,784	2	0.02	22.819 (1.095 – 475.336)	19.183	1.0000	
Chloranil	0	1	159,180	18,161,785	1	0.01	38.032 (1.549 – 933.643)	28.774	1.0000	
Thymoquinone	0	1	159,180	18,161,785	1	0.01	38.032 (1.549 – 933.643)	28.774	1.0000	
Mitoquinone	0	1	159,180	18,161,785	1	0.01	38.032 (1.549 – 933.643)	28.774	1.0000	
Triaziquone	0	1	159,180	18,161,785	1	0.01	38.032 (1.549 – 933.643)	28.774	1.0000	
Diaziquone	0	1	159,180	18,161,785	1	0.01	38.032 (1.549 – 933.643)	28.774	1.0000	

Expected co-reports (E) were calculated under the assumption of independence as E = (a + b)(a + c)/N using the uncorrected marginal totals and are presented for descriptive purposes only; no additional threshold based solely on the observed count (a) was applied. *Fisher's exact $p < 0.05$. A lower reporting disproportionality was defined as an ROR < 1, an upper 95% confidence limit < 1, and Fisher's exact $p < 0.05$.

Supplementary Table S5. Observed and expected breast cancer-related co-report counts and reporting disproportionality for vitamin K types and homologs after excluding reports with breast cancer-related indication terms recorded in the INDI table.

VK Type	a	b	c	d	Expected E	ROR (95% CI)	PRR	p-value	
VK	42	15,136	128,393	15,911,630	121.42	0.348 (0.257 - 0.470)	0.350	<0.0001	*
VK1	33	12,358	128,402	15,914,408	99.12	0.336 (0.239 - 0.472)	0.338	<0.0001	*
VK2	8	2,101	128,427	15,924,665	16.87	0.502 (0.256 - 0.984)	0.504	0.0268	*
MK-n	2	553	128,433	15,926,213	4.44	0.560 (0.162 - 1.940)	0.562	0.3398	
MK-4	6	1,336	128,429	15,925,430	10.74	0.603 (0.279 - 1.303)	0.605	0.1676	
MK-7	0	217	128,435	15,926,549	1.74	0.285 (0.018 - 4.572)	0.287	0.4270	
VK3	1	749	128,434	15,926,017	6.00	0.248 (0.050 - 1.232)	0.250	0.0367	*
VK4	0	66	128,435	15,926,700	0.53	0.932 (0.058 - 15.063)	0.933	1.0000	

Total number of reports: n = 16,055,201; total number of breast cancer-related reports: 128,435. E denotes the expected number of co-reports under the assumption of independence, calculated as E = (a + b)(a + c)/N using the uncorrected marginal totals. CI, confidence interval. *Fisher's exact $p < 0.05$. A lower reporting disproportionality was defined as a reporting odds ratio (ROR) < 1, an upper 95% confidence limit < 1, and Fisher's exact $p < 0.05$.
